# Dyspnea and dystussia in Parkinson’s disease: patient-reported prevalence and determinants

**DOI:** 10.1007/s00415-025-13008-0

**Published:** 2025-03-22

**Authors:** Veerle A. van de Wetering-van Dongen, Maarten J. Nijkrake, Philip J. van der Wees, Joanna IntHout, S. K. L. Darweesh, Bastiaan R. Bloem, Johanna G. Kalf

**Affiliations:** 1 Department of Rehabilitation, Center of Expertise for Parkinson and Movement Disorders, Radboud University Medical Center, Donders Institute for Brain, Cognition and Behaviour, 6500 HB, P.O. Box 9101 (internal code 898), Nijmegen, The Netherlands; 2 Department of Rehabilitation and IQ Healthcare, Radboud University Medical Center, Radboud Institute for Health Sciences, Nijmegen, The Netherlands; 3 Department for Health Evidence, Radboud University Medical Center, Radboud Institute for Health Sciences, Nijmegen, The Netherlands; 4 Department of Neurology, Center of Expertise for Parkinson and Movement Disorders, Radboud University Medical Center, Donders Institute for Brain, Cognition and Behaviour, Nijmegen, The Netherlands

**Keywords:** Parkinson’s disease, Respiratory dysfunction, Breathing, Dyspnea, Pathophysiology, Screening

## Abstract

**Background:**

The prevalence of respiratory dysfunction in PD is unknown and a better understanding of determinants contributing to respiratory dysfunction is important to facilitate early recognition and treatment.

**Objective:**

To examine the prevalence and determinants of self-reported symptoms of respiratory dysfunction among people with PD.

**Methods:**

In a cross-sectional study, we administered a self-completed questionnaire among a sample of 939 persons with PD. Respiratory dysfunction was defined as experiencing at least one of the following symptoms: breathing difficulties, breathlessness/shortness of breath, tightening of the chest, frequent throat clearing, frequent coughing, or coughing difficulties. A principal component analysis (PCA) was used to define composite constructs of respiratory dysfunction. The association with participant-reported determinants was assessed using multivariable logistic regression models (with adjustment for pulmonary diseases and COVID-19 symptoms).

**Results:**

The overall prevalence rate of respiratory dysfunction was 44% in persons with PD (42% after excluding pulmonary diseases or COVID-19). The PCA resulted in two constructs of respiratory dysfunction: ‘dyspnea’ and ‘dystussia’ (an impaired cough response), which together explained 68% of the total variance. Female sex (OR = 1.39), higher BMI kg/m^2^ (OR = 1.04), longer disease duration (OR = 1.35), greater self-reported rigidity (OR = 1.16), previous pulmonary disease(s) (OR = 7.12), and anxiety (OR = 1.04) were independently associated with ‘dyspnea’. Pulmonary disease(s) (OR = 1.81), COVID-19 symptoms (OR = 2.20), swallowing complaints (OR = 1.48), and speech complaints (OR = 1.02) were independently associated with ‘dystussia’.

**Conclusions:**

Dyspnea and dystussia are common manifestations of respiratory dysfunction among people with PD and deserves more awareness in clinical practice. A proactive screening for the determinants of dyspnea and dystussia may contribute to earlier recognition and treatment of respiratory dysfunction.

**Supplementary Information:**

The online version contains supplementary material available at 10.1007/s00415-025-13008-0.

## Introduction

Although the presence of respiratory symptoms in PD was already described by James Parkinson two centuries ago [1], it is still a poorly understood symptom of Parkinson’s disease (PD) [2]. Both central and peripheral factors are possible mechanisms contributing to respiratory dysfunction in PD [3, 4]. Autonomic dysfunction, damage to the vagal nerve and brainstem dysregulation are suggested as central mechanisms influencing the central drive of ventilation in PD [5, 6]. Suggested peripheral contributors of respiratory dysfunction include laryngeal muscle dysfunction, chest wall rigidity, postural deformities and respiratory muscle weakness [7, 8]. Another important deficit of airway protection is an impaired cough response, also known as dystussia [9]. Unsafe swallowing (dysphagia) combined with dystussia is a risk factor for aspiration pneumonia, which in turn is a strong predictor of death in people with PD [10, 11].

It recently became clear that alterations in the respiratory system, as measured using respiratory function tests or oscillometry, can already present early in the course of PD [12–14]. Less is known about how these early occurring alterations in respiratory function tests progress towards late-stage complications such as pneumonia, and how these initial alterations impact on daily functioning in people with PD. A qualitative study explained which respiratory symptoms people with PD notice themselves and how these impact on their daily functioning. A perceived loss in the regulation of breathing, episodes of breathlessness, as well as changes in coughing were causing discomfort, frustration, speech complaints, avoidance of (social) activities or hospitalization [15]. Early recognition of respiratory dysfunction in PD seems important, aiming to timely start respiratory training or evaluate dopaminergic medication, and as a result to enhance social participation and to prevent complications such as pneumonia or hospitalization [16, 17]. However, little is known about the determinants that are associated with respiratory dysfunction in people with PD to facilitate this early recognition. In addition, the prevalence of respiratory dysfunction remains unclear, with widely differing figures being reported in the literature [18–20]. Therefore, this study aimed to examine the prevalence rate of self-reported symptoms of respiratory dysfunction in PD and to identify the determinants that are independently associated with respiratory dysfunction, in a large sample of people with PD.

## Methods

### Study sample and setting

This cross-sectional study was embedded within the PRoactive and Integrated Management and Empowerment in people with Parkinson Disease—Netherlands (PRIME-NL) study. PRIME-NL is a prospective observational study among people with Parkinson disease and their caregivers [21]. The PRIME-NL study is conducted in two regions within The Netherlands: the PRIME Parkinson care region, including four community hospitals that collaborate directly with the Radboud university medical center, and the usual care region, including 60 community hospitals outside of the PRIME region. Patients were recruited through the Parkinson Vereniging (Dutch Parkinson’s association), ParkinsonNEXT database, and through neurologists in the PRIME Parkinson care region. Participants represented a broad spectrum of people with Parkinson’s disease. Inclusion criteria were confirmed clinical diagnosis of PD by the general practitioner or neurologist and having visited the neurology outpatient clinical of a community hospital center at least once during the year. The PRIME-NL study was approved by the Ethics Committee of the Radboud University Medical Center. All participants provided digital or written informed consent before inclusion in the PRIME-NL study.

### Study assessments

The data used in this study were collected during the baseline measurements of the PRIME-NL study and took place between January and December 2020. Data were gathered digitally through self-administered questionnaires, and in case this was not possible, participants were offered to use either a paper-based self-administration or a telephone-based administration.

#### Respiratory dysfunction

To identify respiratory dysfunction, six binary (yes/no) questions about respiratory symptoms were added to the questionnaire-based assessments of the PRIME study. Currently, there is a lack of a clinical measurement tool to establish respiratory dysfunction. In our study, participants were asked to answer the following questions: (1) did you experience breathing difficulties last month? (2) Did you experience breathlessness or a shortness of breath last month? (3) Did you experience tightening in the chest last month? (4) Did you often have to clear your throat last month? (5) Did you feel like you frequently needed to cough last month? (6) Did you experience difficulties with coughing up phlegm?

Our qualitative study found different descriptors of respiratory symptoms experienced by people with PD. People described a loss of breathing automatism and episodes of breathlessness or a rapid, shallow breathing pattern [15]. Other studies also described this as dyspnea, including for example: “suffering from breathlessness”, “trouble breathing normally”, “my breathing feels rapid” and “my breathing feels tight” [22, 23]. Questions 1, 2 and 3 were based on these findings.

Our qualitative study also described a decreased cough strength and frequent coughing experienced by people with PD [15]. A decreased cough sensitivity and impaired efficacy of cough was also found in other studies [24–26]. Questions 4, 5 and 6 were based on these findings.

To determine the prevalence rate of respiratory dysfunction, we considered each person that answered ‘yes’ on one of the six questions as having respiratory dysfunction. We deliberately set the cutoff low to decrease the risk for underestimation of symptoms [22]. To better understand the possible triggers for respiratory dysfunction, we also added the following two items to the questionnaire-based assessment: respiratory symptoms related to physical exertion (yes/no), and respiratory symptoms related to intake of antiparkinsonian medication (yes/no).

#### Determinants

We included the following potential patient-reported determinants from the main database which could contribute to respiratory dysfunction.

##### Sociodemographic data

The following sociodemographic data of people with Parkinson’s disease were selected for this study: sex, age at diagnosis, time since first symptoms (less than 1 year, 1–3 years, 3–5 years, 5–10 years, > 10 years), smoking (yes/no), smoking history (yes/no), coronary disease (yes/no), pulmonary diseases (yes/no) and body mass index in kg/m^2^ (BMI).

##### Self-reported motor symptoms

The motor symptoms tremor, bradykinesia left and right, rigidity and freezing were scored by the participants on a range from 1 (no symptoms) to 10 (severe symptoms).

##### Swallowing function

Swallowing function was evaluated using the Movement Disorders Society Unified Parkinson Disease Rating Scale (MDS-UPDRS) part II (motor experiences of daily living) items 2.2 saliva and drooling, and 2.3 chewing and swallowing [27]. These items were scored on a 5-point Likert scale ranging from 0 (normal) to 4 (severe).

##### Quality of life, subscale communication

The sum score of the dimension communication from the Parkinson’s Disease Questionnaire (PDQ-39) was used to assess health-related quality of life in people with PD [28].

##### COVID-19 symptoms

All participants were asked whether they experienced symptoms related to COVID-19 (e.g., coughing, fever, dyspnea, a sore throat) even without having a positive test. The experience of COVID-19 symptoms was dichotomized (0 = no symptoms, 1 = symptoms).

##### State-trait anxiety inventory (STAI)

The STAI questionnaire and was used to evaluate anxiety. The cutoff score for separating those with a mental disorder from those without was ≥ 54 for elderly people [29].

### Statistical analysis

Descriptive statistics were used to describe the participants’ characteristics and to calculate the prevalence rate of respiratory dysfunction in absolute numbers and percentage. In case of missing data at random, multiple imputation was used, to impute missing data [30]. Ten imputed datasets were created, and the pooled dataset was used for further analysis.

We defined respiratory dysfunction as our dependent variable in terms of the six questions mentioned earlier in our methods about respiratory symptoms. A principal component analysis (PCA) was conducted to cluster the six questions into a minimum number of subdomains to simplify the clinical interpretation. The subdomains were clustered by their factor structure and contain more information than only the outcome of the separate items. We used a scree plot and an eigenvalue of ≥ 1 to determine the number of subdomains. Since we expected that the six questions were related, we used oblique rotation (direct oblimin) to optimize configuration on factors, allowing for maximum amount of non-orthogonality (Delta = 0). The internal consistency of the factors was measured using Cronbach’s alpha (α) and we took 0.70 as a minimum of acceptable internal consistency [31].

Collinearity was checked using the Pearson’s correlation coefficient. The determinants were dichotomized and examined in both univariate and multivariable logistic regression models. The following steps were taken to develop these models. First, univariate analyses were conducted for all potential determinants preceding the outcome (potentially causal), generating odds ratios with 95% confidence intervals. Second, a multivariable regression model was created with all potential determinants to determine the independent determinants related to respiratory dysfunction in people with PD. As a sensitivity analysis, we excluded people diagnosed with coronary artery diseases, pulmonary diseases, or COVID-19 symptoms (presumably mediators), which might be independent determinants and/or in the causal pathway of other preceding determinants. Data analysis was performed using IBM SPSS Statistics, version 25.

## Results

In total, 939 people with PD completed the questionnaires. Their characteristics are shown in Table 1 (*n* = 939; six participants were excluded due to missing items on almost all variables). All continuous variables were reasonably normally distributed. In total, 60.8% of the participants were men and the mean age was 70 years (SD 8); 22.6% of all the participants had a coronary artery disease and 9.9% a pulmonary disease.

**Table 1:** Characteristics of the study population (*n* = 939)

Prevalence respiratory symptoms; number (%)	
Breathing difficulties	140 (14.9%)
Shortness of breath or breathlessness	149 (15.9%)
Tightening in the chest	131 (14.0%)
Often have to clear your throat	280 (29.8%)
Frequently needed to cough	170 (18.1%)
Coughing difficulties	109 (11.6%)
Age in years; mean (SD)	70 (8)
Men; number (%)	571 (60.8%)
Nationality; number (%)	
Dutch	930 (99.0%)
Other	9 (1.0%)
Marital status, *n* (%)	
Married	729 (77.6%)
Living with partner	62 (6.6%)
Divorced	35 (3.7%)
Widow/widower	58 (6.2%)
Single/unmarried	50 (5.3%)
Long distance relationship	5 (0.5%)
Living situation, number (%)	
Living on my own	132 (14.1%)
Living with my partner	720 (76.7%)
Living with my partner and children	64 (6.8%)
Living in an institution, i.e., nursing home	9 (0.9%)
Living independently, but receiving ambulatory support	6 (0.6%)
Living together with another family member	8 (0.9%)
Working status, *n* (%)	
Paid	115 (12.2%)
Not paid	824 (87.8%)
Time since first symptoms; number (%)	
Less than 1 year	21 (2.2%)
1–3 years	192 (20.4%)
3–5 years	181 (19.3%)
5–10 years	310 (33.0%)
> 10 years	235 (25.0%)
Body mass index in kg/m^2^; mean (SD)	26 (4)
Missing	16 (1.7%)
Smoking; number (%)	
Yes	27 (2.9%)
No	912 (97.1%)
Previous smoking; number (%)	
Yes	533 (56.8%)
No	406 (43.2%)
Hospitalization last year; number (%)	
No	775 (82.5%)
One time	128 (13.6%)
2–3 times	32 (3.4%)
> 3 times	4 (0.4%)
Pneumonia last year; number (%)	
No	908 (96.7%)
Yes	31 (3.3%)
One time	27 (2.9%)
2–3 times	4 (0.4%)
Treatment antibiotics	29 (3.1%)
Hospitalization	11 (1.2%)
For 2–3 days	2 (0.2%)
> 3 days	9 (1.0%)
Comorbidities; number (%)	
Coronary artery diseases	212 (22.6%)
Angina pectoris	28 (3.0%)
Heart failure	16 (1.7%)
Myocardial infarction	37 (3.9%)
Heart arrhythmia	95 (10.1%)
Stroke	26 (2.8%)
Pulmonary diseases	93 (9.9%)
Asthma	44 (4.7%)
COPD	40 (4.3%)
Pulmonary hypertension	2 (0.2%)
Musculoskeletal disorders	270 (28.8%)
Arthrosis	181 (19.3%)
Contractures	10 (1.1%)
Pressure ulcers	0 (0%)
Osteoporosis	43 (4.6%)
Neuropsychiatric disorders	80 (8.5%)
Anxiety disorder	37 (3.9%)
Dementia	5 (0.5%)
Depression	37 (3.9%)
Addiction	7 (0.7%)
Endocrine or metabolic disorders	91 (9.7%)
Diabetes mellitus	49 (5.2%)
Kidney failure	5 (0.5%)
Liver disease	2 (0.2%)
Thyroid disease	35 (3.7%)
Cancer	82 (8.7%)
Breast cancer	9 (1.0%)
Colon cancer	14 (1.5%)
Skin cancer	23 (2.4%)
Lung cancer	1 (0.1%)
Prostate cancer	29 (3.1%)
COVID-19 symptoms; number (%)	844 (89.9%)
Yes	155 (16.5%)
No	689 (73.4%)
Missing	95 (10.1%)
Respiratory symptoms related to medication; number (%)	31 (3.3%)
ON-state and annoying dyskinesia	8 (0.9%)
ON-state and not annoying dyskinesia	3 (0.3%)
ON-state without dyskinesia	1 (0.1%)
OFF-state	12 (1.3%)
When medication started to work	9 (1.0%)
When medication started to wearing out	11 (1.2%)
Respiratory symptoms related to physical exertion; number (%)	173 (18.4%)

The following variables included missing values: COVID-19 symptoms (10.1%), BMI (1.7%), self-reported freezing (1.6%), self-reported rigidity (1.5%), self-reported bradykinesia left (1.2%), self-reported bradykinesia right (0.5%) and self-reported tremor (0.6%).

### Respiratory dysfunction

A PCA was performed with the six respiratory symptoms using oblique rotation (direct oblimin) (see Appendix 1). Two factors had eigenvalues > 1 and together they explained 68.4% of the variance, so we divided respiratory dysfunction in two subdomains (see Appendix 1, Table 3). The factor loadings after rotation showed that the first subdomain ‘dyspnea’ represented the symptoms ‘breathing difficulties’, ‘breathlessness or shortness of breath’ and ‘tightening in the chest’. The second subdomain ‘dystussia’ represented the symptoms ‘often have to clear their throat’, ‘frequently needed to cough’ and ‘coughing difficulties’ (Table 2). Cronbach’s alpha (α) was 0.82 for the subdomain ‘dyspnea’ and 0.70 for the subdomain ‘dystussia’, which was considered acceptable.

**Table 2:** Rotated factor loadings (*n* = 939)

Pattern matrix^a^	Component	
	1	2
Breathing difficulties	**0.896**	0.019
Shortness of breath or breathlessness	**0.900**	−0.058
Tightening in the chest	**0.765**	0.043
Often have to clear your throat	−0.032	**0.816**
Frequently needed to cough	0.026	**0.794**
Coughing difficulties	0.010	**0.777**
Eigenvalues	2.7	1.4
% of variance	44.8%	23.7%
α	0.82	0.70

Extraction method: principal component analysis

Rotation method: Oblimin with Kaiser normalization

^a^Rotation converged in 4 iterations

Bold values indicates loadings of component 1 (dyspnea) and component 2 (dystussia)

### Prevalence rate of respiratory dysfunction

Four hundred and sixteen (44.3%) out of 939 people with PD experienced at least one of the six respiratory symptoms. The prevalence rate of each symptom is shown in Table 1. At least one item of the subdomain dyspnea was experienced by 22.7%, at least one item of the subdomain dystussia was experienced by 35.1%, and 13.5% of the participants confirmed at least one item on both the subdomains. When excluding people with a pulmonary disease (*n* = 93) from the total dataset, 354 people (41.8%) presented at least one respiratory symptom. After excluding people with COVID-19 symptoms (*n* = 174), the prevalence rate of people presented one respiratory symptom reduced to 41.7% (*n* = 318).

Respiratory symptoms were related to intake of levodopa medication in 3.3% (*n* = 31) of participants and symptoms were experienced either in “off-state” (*n* = 12), “on-state with dyskinesia” (*n* = 11), “on state without dyskinesia” (*n* = 1), and “in a biphasic pattern” (*n* = 20). In addition, 18.4% (*n* = 173) of all the participants reported that their respiratory symptoms were related to physical exertion.

### Directed acyclic graph (DAG)

**Fig. 1:**
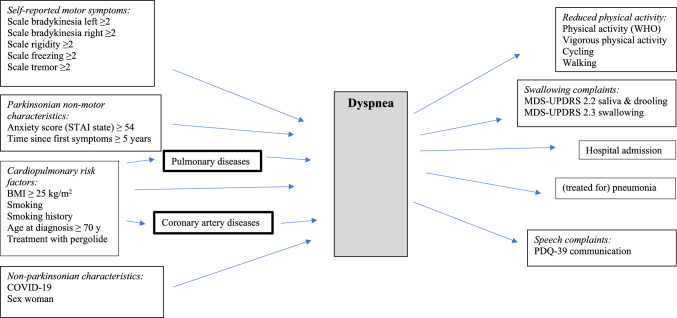
Directed acyclic graph (DAG) for dyspnea

**Fig. 2:**
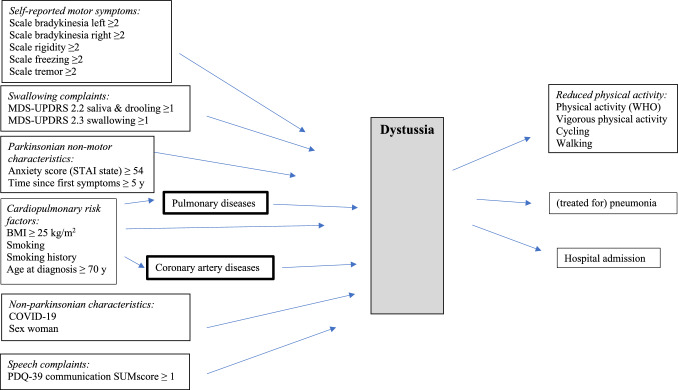
Directed acyclic graph (DAG) for dystussia

The DAGs in Figs. 1 and 2 illustrate the likely pathways for subdomain ‘dyspnea’ and subdomain ‘dystussia’. Multicollinearity was assessed and showed that any determinant that was included in the model had a Pearson’s correlation coefficient *r* < 0.3. The Parkinsonian self-reported motor symptoms, anxiety score, time since first symptoms, cardiopulmonary risk factors (BMI, smoking (history), age at diagnosis, treatment with pergolide), COVID-19, and sex were assumed to have a direct effect on ‘dyspnea’ [7]. Reduced physical activity, hospital admission, (treated for a) pneumonia, swallowing and speech complaints are considered as potential results of dyspnea. The self-reported motor symptoms, anxiety score, time since first symptoms, cardiopulmonary risk factors (BMI, smoking (history), age at diagnosis, treatment with pergolide), COVID-19, sex, swallowing and speech complaints were assumed to have a direct effect on dystussia [32]. Reduced physical activity, hospital admission and (treatment for a) pneumonia are depicted as potential results of dystussia [33]. The cardiopulmonary risk factors potentially could be mediated by pulmonary diseases or coronary artery diseases. To verify whether these two were mediators, we performed a separate analysis in which participants with pulmonary or coronary artery diseases were excluded.

### Univariate analyses

#### Subdomain dyspnea

Table 3 summarizes the univariate analyses in 213 people who had experienced ‘dyspnea’ compared to 726 people without ‘dyspnea’. People with dyspnea were more often women, had a longer disease duration (> 5 years) and a lower age at the moment of diagnosis. People with dyspnea also experienced severe motor symptoms (bradykinesia and rigidity). Of these two self-reported motor symptoms, rigidity showed the highest association with an odds ratio (OR) of 2.34 [95% confidence interval (CI) 1.15–4.48] for dyspnea. Having a pulmonary disease in addition to PD was more likely in the dyspnea group and was associated with an OR = 5.68 (95% CI 3.64–8.87) for dyspnea. Higher anxiety levels (STAI state ≥ 54) were associated with an OR = 3.61 (95% 2.24–5.81) for dyspnea. A BMI ≥ 25 (OR 1.54; 95% CI 1.12–2.11) was associated with higher odds for dyspnea as well. People treated with pergolide were more likely to experience dyspnea, although the absolute number of people treated with pergolide was very small in this population. No significant differences between the groups were found for smoking, smoking history, coronary artery diseases, COVID-19 symptoms, freezing and tremor.

**Table 3:** Results of univariate logistic regression for subdomain ‘dyspnea’

Dyspnea	Dyspnea (*n* = 213), *n* (%)	No dyspnea (*n* = 726), *n* (%)	Odds ratio (95% CI^a^)	*p* value
Self-reported motor symptoms				
Scale bradykinesia left ≥ 2	179 (84.0)	548 (75.5)	1.71 (1.14–2.56)	0.01
Scale bradykinesia right ≥ 2	176 (82.6)	537 (74.0)	1.68 (1.13–2.48)	0.01
Scale rigidity ≥ 2	204 (95.8)	657 (90.5)	2.34 (1.15–4.48)	0.02
Scale freezing ≥ 2	99 (46.5)	288 (39.7)	1.32 (0.97–1.80)	0.08
Scale tremor ≥ 2	211 (99.1)	714 (98.3)	1.77 (0.40–7.99)	0.46
Parkinsonian nonmotor characteristics				
Anxiety score ≥ 54 (STAI state^b^)				
Time since first symptoms	37 (17.4)	40 (5.5)	3.61 (2.24–5.81)	< 0.001
< 1 year	4 (1.9)	17 (2.3)		
1–3 years	19 (8.9)	173 (23.8)		
3–5 years	39 (18.3)	142 (19.6)		
5–10 years	77 (36.2)	233 (32.1)		
> 10 years	74 (34.7)	161 (22.2)		
Time since first symptoms ≥ 5 years	151 (70.9)	394 (54.3)	2.05 (1.48–2.85)	< 0.001
Cardiopulmonary risk factors				
Pulmonary diseases	53 (24.9)	40 (5.5)	5.68 (3.64–8.87)	< 0.001
Coronary artery diseases	53 (24.9)	159 (21.9)	1.18 (0.83–1.69)	0.36
BMI^c^ ≥ 25 kg/m^**2**^	126 (59.2)	353 (48.6)	1.54 (1.12–2.11)	0.01
Age at diagnosis ≤ 70 years	173 (81.2)	533 (73.4)	1.57 (1.07–2.29)	0.02
Smoking	9 (4.2)	18 (2.5)	1.74 (0.77–3.92)	0.19
Smoking history	123 (57.7)	409 (56.5)	1.06 (0.78–1.46)	0.70
Treatment with pergolide	14 (6.6)	6 (0.8)	1.03 (0.94–1.13)	0.53
Non-parkinsonian characteristics				
COVID-19	46 (21.6)	128 (17.8)	1.28 (0.87–1.90)	0.22
Sex woman	98 (46.0)	270 (37.2)	1.44 (1.06–1.96)	0.02

^a^Confidence interval

^b^State-trait anxiety inventory

^c^Body mass index

### Subdomain ‘dystussia’

Table 4 summarizes the univariate analysis in 330 people who had experienced ‘dystussia’ compared to 609 people without any coughing symptoms. People experiencing dystussia had severe motor symptoms (bradykinesia, rigidity and freezing) and a disease duration ≥ 5 years (OR = 1.30; 95% CI 0.99–1.71). Having a pulmonary disease, coronary disease or smoking history was associated with dystussia. In addition, higher anxiety levels (STAI state ≥ 54) were associated with dystussia (OR = 2.01; 95% CI 1.26–3.22). COVID-19 symptoms were assessed in 26.1% of people experiencing dystussia compared to 14.4% in people without any coughing symptoms (OR = 2.08; 95% CI 1.46–2.97). Both swallowing complaints (OR = 2.83; 95% CI 2.14–3.75) and speech complaints (OR 2.84; 95% CI 1.95–4.15) were more frequently assessed in the dystussia group and were associated with dystussia.

**Table 4:** Results of univariate logistic regression for subdomain ‘dystussia’

Dystussia	Dystussia (*n* = 330) *n* (%)	No dystussia (*n* = 609) *n* (%)	Odds ratio (95% CI^a^)	*p* value
Self-reported motor symptoms				
Scale bradykinesia left ≥ 2	272 (82.4)	455 (74.7)	1.59 (1.13–2.22)	0.01
Scale bradykinesia right ≥ 2	263 (79.7)	450 (73.9)	1.39 (1.00–1.92)	0.05
Scale rigidity ≥ 2	309 (93.6)	552 (90.6)	1.51 (0.90–2.55)	0.12
Scale freezing ≥ 2	149 (45.2)	238 (39.1)	1.28 (0.98–1.68)	0.07
Scale tremor ≥ 2	324 (98.2)	601 (98.7)	0.72 (0.25–2.09)	0.54
Parkinsonian nonmotor characteristics				
Anxiety score ≥ 54 (STAI state^b^)	39 (11.8)	38 (6.2)	2.01 (1.26–3.22)	0.003
Time since first symptoms				
< 1 year	5 (1.5)	16 (2.6)		
1–3 years	46 (13.9)	146 (24.0)		
3–5 years	74 (22.4)	107 (17.6)		
5–10 years	113 (34.2)	197 (32.3)		
> 10 years	92 (27.9)	143 (23.5)		
Time since first symptoms ≥ 5 years	205 (62.1)	340 (55.8)	1.30 (0.99–1.71)	0.06
Cardiopulmonary risk factors				
Pulmonary diseases	44 (13.3)	49 (8.0)	1.76 (1.14–2.71)	0.01
Coronary artery diseases	93 (28.2)	119 (19.5)	1.62 (1.18–2.21)	0.003
BMI^c^ ≥ 25 kg/m^**2**^	168 (50.9)	311 (51.2)	0.99 (0.76–1.30)	0.96
Age at diagnosis ≤ 70 years	244 (73.9)	462 (75.9)	0.90 (0.66–1.23)	0.52
Smoking	7 (2.1)	20 (3.3)	0.64 (0.27–1.53)	0.31
Smoking history	198 (60.0)	335 (55.0)	1.23 (0.93–1.61)	0.15
Treatment with pergolide	10 (3.0)	10 (1.6)	1.00 (0.92–1.09)	0.93
Non-parkinsonian characteristics				
Covid-19 symptoms	86 (26.1)	88 (14.4)	2.08 (1.46–2.97)	< 0.001
Sex woman	122 (37.0)	246 (40.4)	0.87 (0.66–1.14)	0.31
Swallowing complaints				
MDS-UPDRS^d^ 2.2 saliva and drooling ≥ 1	229 (69.4)	340 (55.8)	1.79 (1.35–2.38)	< 0.001
MDS-UPDRS 2.3 swallowing ≥ 1	172 (52.1)	169 (27.8)	2.83 (2.14–3.75)	< 0.001
Speech complaints				
PDQ-39^e^ communication ≥ 1	291 (88.2)	441 (72.4)	2.84 (1.95–4.15)	< 0.001

^a^Confidence interval

^b^State-trait anxiety inventory

^c^Body mass index

^d^Movement Disorder Society unified Parkinson’s disease rating scale

^e^Parkinson’s Disease Questionnaire-39

### Multivariable analyses

#### Subdomain ‘dyspnea’

Table 5 shows the multivariable logistic regression analysis for subdomain ‘dyspnea’. Model 1 shows that ‘dyspnea’ is significantly higher among women [odds ratio (OR) = 1.39, 95% confidence interval (CI) 0.98–1.99], people with a higher BMI per kg/m^2^ (OR 1.04, 95% CI 1.00–1.09), a longer disease duration in years (OR 1.35, 95% CI 1.13–1.61), more rigidity (OR 1.16, 95% CI 1.06–1.27), and more anxiety (OR 1.04, 95% CI 1.02–1.06). Having a pulmonary disease was also associated with an odds ratio of 7.12 (95% CI 4.34–11.67) for dyspnea.

**Table 5:** Multivariable models for subdomain ‘dyspnea’

Dyspnea	Model 1 (all participants)	Model 2 (participants without pulmonary diseases)	Model 3 (participants without COVID-19 symptoms)
	OR (95% CI^b^)	OR (95% CI)	OR (95% CI)
Self-reported motor symptoms			
Scale bradykinesia left	1.02 (0.94–1.11)	1.00(0.92–1.11)	1.02 (0.93–1.13)
Scale bradykinesia right	1.00 (0.91–1.09)	1.00(0.91–1.10)	1.03 (0.93–1.14)
Scale rigidity	**1.16** (**1.06–1.27)**	**1.20 (1.09–1.33)**	**1.15 (1.04–1.27)**
Scale freezing	0.98 (0.90–1.05)	0.96 (0.89–1.04)	0.98 (0.90–1.07)
Parkinsonian nonmotor characteristics			
Anxiety score (STAI state^c^)	**1.04 (1.02–1.06)**	**1.04 (1.03–1.06)**	**1.04 (1.02–1.06)**
Time since first symptoms	**1.35 (1.13–1.61)**	**1.40 (1.15–1.70)**	**1.25 (1.03–1.52)**
Cardiopulmonary risk factors			
Pulmonary diseases	**7.12 (4.34–11.67)**		**6.71 (3.86–11.68)**
BMI^d^ (kg/m^2^)	**1.04 (1.00–1.09)**	**1.05 (1.01–1.10)**	**1.05 (1.0–1.09)**
Age at diagnosis (per year)	0.99 (0.97–1.01)	0.99 (.96–1.01)	0.98(0.96–1.00)
Smoking	1.35 (0.55–3.32)	1.17 (0.45–3.06)	0.76(0.24–2.43)
Non-parkinsonian characteristics			
COVID-19	1.12 (0.72–1.75)	1.07 (0.66–1.74)	
Sex woman	**1.39 (0.98–1.99)**	**1.62 (1.11–2.35)**	1.40 (0.95–2.07)

Model 1: multivariable logistic regression including all determinants that preceding dyspnea. Model 2: same variables as model 1, people diagnosed with a pulmonary disease were excluded

^a^Odds ratio

^b^Confidence interval

^c^State-trait anxiety inventory

^d^Body mass index

Bold values indicates Significant of odds ratios

As a sensitivity analysis, people diagnosed with a pulmonary disease (model 2, *n* = 93) or people that had COVID-19 symptoms (model 3, *n* = 174) were excluded from the dataset. Despite the exclusion of participants with pulmonary diseases (model 2) or participants with COVID-19 symptoms (model 3), results showed the same determinants with similar odds ratios.

### Subdomain ‘dystussia’

Table 6 shows the multivariable logistic regression analysis for the subdomain ‘dystussia’. Model 1 on all participants included the determinants pulmonary disease (OR 1.81, 95% CI 1.14–2.88), COVID-19 symptoms (OR 2.20, 95% CI 1.51–3.20), swallowing complaints (OR 1.48, 95% CI 1.24–1.77) and speech complaints (OR 1.02, 95% CI 1.01–1.03). After the exclusion of people diagnosed with pulmonary diseases, coronary artery diseases or COVID-19 symptoms, model 2, 3 and 4 resulted in similar determinants and odds ratios.

**Table 6:** Multivariable models for subdomain ‘dystussia’

Dystussia	Model 1 (all participants)	Model 2 (participants without pulmonary diseases)	Model 3 (participants without coronary artery diseases)	Model 4 (participants without COVID-19 symptoms)
	OR^a^ (95% CI^b^)	OR (95% CI)	OR (95% CI)	OR (95% CI)
Self-reported motor symptoms				
Scale bradykinesia left	1.01 (0.93–1.09)	1.01 (0.93–1.09)	0.98 (0.90–1.07)	0.98 (0.90–1.07)
Scale bradykinesia right	0.97 (0.90–1.05)	0.98 (0.90–1.06)	0.99 (0.91–1.09)	1.00 (.091–1.10)
Scale rigidity	1.05 (0.98–1.13)	1.07 (0.99–1.16)	1.09 (1.00–1.19)	1.07 (0.98–1.17)
Scale freezing	0.99 (0.92–1.06)	0.97 (0.91–1.05)	0.96 (0.88–1.04)	0.97 (0.90–1.05)
Parkinsonian nonmotor characteristics				
Anxiety score (STAI state^c^)	1.01 (0.99–1.02)	1.01 (1.00–1.03)	1.01 (0.99–1.02)	1.01 (0.99–1.03)
Time since first symptoms	1.01 (0.88–1.16)	1.00 (0.87–1.16)	1.02 (0.87–1.20)	0.98 (0.84–1.15)
Cardiopulmonary risk factors				
Pulmonary disease	**1.81 (1.14–2.88)**		1.67 (0.96–2.92)	1.58 (0.93–2.67)
Coronary artery disease	1.41 (1.01–1.97)	1.37 (0.96–1.96)		1.35 (0.92–1.97)
Smoking history	1.10 (0.81–1.48)	1.15 (0.84–1.57)	1.13 (0.80–1.58)	1.09 (0.78–1.54)
Non-parkinsonian characteristics				
COVID-19	**2.20 (1.51–3.20)**	**2.04 (1.36–3.04)**	**2.10 (1.36–3.24)**	
Swallowing complaints				
MDS-UPDRS^d^ 2.2 saliva and drooling	1.06 (0.93–1.21)	1.06 (0.92–1.22)	1.08 (0.93–1.25)	1.11 (0.96–1.29)
MDS-UPDRS 2.3 swallowing	**1.48 (1.24–1.77)**	**1.41 (1.17–1.70)**	**1.59 (1.29–1.94)**	**1.49 (1.21–1.83)**
Speech complaints				
PDQ-39^e^ communication	**1.02 (1.01–1.03)**	**1.02 (1.01–1.03)**	**1.02 (1.01–1.03)**	**1.02 (1.01–1.03)**

Model 1: multivariable logistic regression including all determinants that preceding dystussia. Model 2: same variables as model 1, people diagnosed with a pulmonary disease were excluded. Model 3: same variables as model 1, people diagnosed with a coronary artery disease were excluded

^a^Odds ratio

^b^Confidence interval

^c^State-trait anxiety inventory

^d^Movement Disorder Society unified Parkinson’s disease rating scale

^e^Parkinson’s Disease Questionnaire-39

Bold values indicates Significant of odds ratios

## Discussion

This study examined the prevalence rate of self-reported symptoms of respiratory dysfunction in PD and showed a prevalence rate of 44% of people with PD experience respiratory dysfunction. Our analyses revealed two internally consistent subdomains that represent respiratory dysfunction, namely dyspnea and dystussia. Moreover, this study identified the determinants that are independently associated with respiratory dysfunction. For dyspnea, the independently associated determinants were female sex, a higher BMI, a longer disease duration, previous pulmonary disease, more rigidity, and experiencing more anxiety. For dystussia, the independently associated determinants were previous pulmonary disease, COVID-19 symptoms, swallowing problems, and speech complaints.

Prevalence rates of respiratory dysfunction in PD in previous studies ranged from 18 to 94% [4, 19]. This wide range can be explained by the use of different definitions for respiratory symptoms and by the heterogeneity of the populations that were studied. Respiratory dysfunction was defined either based on outcomes of pulmonary function tests, dyspnea questions or questionnaires (e.g., MRC dyspnea scale) or by interviewing participants about several respiratory symptoms. Only two studies found a similar prevalence rate of respiratory dysfunction of around 40% [22, 34]. Both studies were considerably smaller than our cohort but used comparable questions to assess respiratory dysfunction. A strength of our study was not only the large sample size, but also the representative nature of the studied population. Specifically, age, sex and ethnicity are comparable with the full PD population [35]. One large study found a prevalence of only 17.8% for respiratory symptoms, but this study included a random population of people with Parkinsonism, and it was unclear how they precisely assessed respiratory symptoms (defined as dyspnea, coughing and stridor) [36].

### Subdomain dyspnea

The origin of dyspnea in humans seems multifactorial including psychological, social, physical and environmental influences [37]. Dyspnea is well known and a prevalent symptom in people diagnosed with a pulmonary disease [38]. Respiratory function tests are normally used to measure respiratory dysfunction objectively, but their relation with the severity of dyspnea perceived by people with a pulmonary condition seems weak [38, 39]. The MRC dyspnea scale is a commonly recommended measurement tool which grades the effect of dyspnea on daily activities in people with pulmonary diseases [40]. The scale consists of statements about perceived breathlessness during physical activities. Remarkably, only 18.3% of the total population in our study experienced respiratory symptoms related to physical exertion. This was much lower than the experience of dyspnea related to physical exertion in many pulmonary diseases, such as COPD [39, 41]. One explanation, supported by a qualitative study asking people with PD for triggers of dyspnea, is that there are more triggers for dyspnea, of which physical (in)activity is only one. Other triggers for dyspnea were stress, anxiety, postural deformities and fatigue [8, 15]. This also raises the questions whether current dyspnea scales, often adopted from the field of pulmonary diseases and often having their focus on physical exertion, are suitable to adequately capture the triggers for dyspnea in PD.

Both motor and nonmotor features seem to be associated with dyspnea in PD. In line with another study, we found a relation with rigidity in PD [4, 10]. It is known that rigidity contributes to postural deformities and chest wall stiffness [42]. In turn, this may contribute to decreased lung volumes and at least partly explain the compromised breathing in people with PD.

Anxiety is a prevalent nonmotor symptom, and in a qualitative study, this was already mentioned by people with PD as a trigger for rapid shallow breathing [15]. We quantified this finding as anxiety was associated with dyspnea in people with PD in our study [43, 44]. Others used the multidimensional dyspnea profile (MDP), an instrument to distinguish sensory and emotional aspects of dyspnea, and showed that dyspnea in PD seems to be related to anxiety and depression [45, 46].

Our findings also showed that respiratory dysfunction seems to increase with disease progression. This finding is in line with other longitudinal studies that also reported a decline in respiratory function parameters with disease progression [3, 10, 12]. For example, Barone et al. reported respiratory symptoms in 9.6% with mild (HY stage = 1) and 30.6% with severe (HY stage = 4–5) Parkinson’s disease [36]. A new finding was that being a woman was associated with development of dyspnea. Increasing evidence shows that PD differs between women and men, which includes among others the fact that women have a higher mortality rate and faster progression of the disease [47]. In addition, there are differences in the respiratory tract between women and men in general, and that men tend to have a better respiratory function [48].

A higher BMI was associated with the development of dyspnea. It is well known that increased body weight decreases lung volumes [49]. Moreover, there is a significant linear relationship between BMI and vital capacity [50].

### Subdomain dystussia

Swallowing and drooling complaints are common in PD and are related to the respiratory system [41]. Cough protects the airway and is important for airway clearance when penetration or aspiration occurs during swallowing [51]. Moreover, earlier research described that a decreased cough sensitivity and cough strength was related to aspiration [25, 52]. Recent studies described an atypical respiratory-swallow coordination (RSC), as people initiate swallows at significantly lower lung volumes, inhale more frequently before and after swallowing and have longer periods of swallow apnea compared to healthy adults [53, 54]. This was associated with the presence of dysphagia and pulmonary aspiration and leads to risks like an aspiration pneumonia. RSC and coughing can be improved by verbal cueing on specific points during tidal breathing, cough skill training or by expiratory muscle strength training [55–58].

Pulmonary diseases were related to dystussia in our study. Coughing problems were not only a symptom of people with PD but was also associated with several pulmonary diseases such as asthma, bronchitis and idiopathic pulmonary fibrosis. [59–61] Chronic cough (cough lasting > 8 weeks) was associated with a reduced psychosocial quality of life [15, 62].

Speech complaints—known as hypokinetic dysarthria with a softer voice as main characteristic—were also associated with dystussia. Voice production highly depends on the respiratory subsystem that needs to provide enough lung volume and expiratory airflow [44, 63–65]. Speech-language therapy guidelines do recommend improving speech outcomes with interventions such as Lee Silverman Voice Treatment (LSVT) and Pitch Limiting Voice Treatment (PLVT), which focus on improving vocal loudness. This suggests that these techniques may also improve expiratory pressure and coughing, but this has not been taking into account yet. At the same time, guidelines might need to include recommendations about respiratory training to increase expiratory strength and resulting in more effective coughing [17, 55, 66].

### Strengths and limitations

A strength of this study is the consideration of causal pathways using the directed acyclic graphs (DAG) approach. A DAG is used to illustrate the origin, causal pathways, and consequences of respiratory dysfunction in people diagnosed with PD [67, 68]. Importantly, variables that might also be a consequence of dyspnea or coughing do not meet the prerequisites for being an associated determinant. Our DAG helped to model the connections and causality by illustrating the sequence of determinants based on the authors’ previous knowledge and assumptions [69].

Another strength is the recruiting strategies of our included population. We used multiple recruiting strategies to arrive at a sample that was representative for the actual PD population based on demographic variables. We included people in different regions and different (academic and community) hospitals in the Netherlands. Our sample represents a mean age of 70 years, 39% woman, 99% native Dutch people which is comparable with the full PD population (with a mean age of 66 years, 39% woman and 97–100% native Dutch people) [35, 70].

Another limitation of this study was that the motor symptoms were assessed using a self-reported questionnaire instead of MDS-UPDRS part III examination. The COVID-19 pandemic, with its combination of lockdowns on the one hand and limited availability of respiratory function tests on the other, made this impossible at that moment. However, subjective examination of motor symptoms at least gave a better understanding about the experienced impact of motor symptoms in our population [71]. Therefore, our findings do restrict the generalizability of our study population in relation to disease severity. For this reason, we cannot draw any conclusions about respiratory dysfunction in relation to disease stages. It seems likely that the people in a late stage of PD were outnumbered in our study and so on the prevalence of respiratory dysfunction in our study should be an underestimation instead of an overestimation. Also, we found that a longer disease duration was associated with dyspnea and that was in line with the findings of another study in which the MDS-UPDRS part III was examined [72].

Another weakness of this study is the interference of the COVID-19 pandemic, as COVID-19 also results in respiratory symptoms. During our study, testing and vaccination for COVID-19 infection was rare. To evaluate possible bias, the participants were asked, at a later date, if they had experienced symptoms related to COVID-19. When we excluded the 163 people with COVID-19 symptoms from the dataset, the prevalence rate of respiratory symptoms and the associated determinants remained similar. We, therefore, feel that COVID-19 did not impact the study results much.

Although we believe that the six questions about respiratory dysfunction were sensitive enough to detect people with PD and respiratory dysfunction, these questions were not part of an established clinical measurement tool. An increasing number of studies recommend including dyspnea and coughing as nonmotor symptoms related to PD [2, 36, 73, 74]. To quantify respiratory dysfunction in PD related to its disability and impact on daily activities, our study generated two relevant subdomains that measured respiratory dysfunction using construct analysis and a DAG approach was developed based on the current evidence. This was a first step to establish the measurement of respiratory dysfunction in clinical practice.

## Conclusion

This study shows that 44% of 939 people with PD experience respiratory dysfunction, described as symptoms of dyspnea or dystussia. Determinants associated with dyspnea were female sex, a higher BMI, a longer disease duration, pre-existing pulmonary disease, more rigidity, and experiencing more anxiety. Determinants associated with dystussia were pre-existing pulmonary disease, COVID-19 symptoms, swallowing problems, and communication difficulties.

The subdomains together with the identified determinants can help clinicians to screen for respiratory dysfunction and to recognize already at an early stage which people with Parkinson’s disease are at risk for developing respiratory dysfunction. Early recognition could help to minimize the impact of respiratory dysfunction on quality of life and social activities. In addition, it could also help to prevent late-stage respiratory complications, such as an aspiration pneumonia. Both dopaminergic medication and non-pharmacological interventions (respiratory training) can improve respiratory function and have the potential to prevent late-stage complications. This presumably requires a multidisciplinary approach and given that multiple factors are related to respiratory dysfunction, a personalized approach as well.

Future research should focus on whether a decrease in pulmonary function and respiratory muscle strength tests is related to the determinants found for respiratory dysfunction in people with PD. The determinants should not only include self-reported items, but also physically examined parameters (e.g., MDS-UPDRS part III). In addition, there is a need for better tools to measure both the signs and symptoms of respiratory dysfunction and their impact on participation in daily life. Finally, more research is needed to determine what would be the right moment to start with respiratory training to prevent late-stage complications such as pneumonia.

## Supplementary Information

Below is the link to the electronic supplementary material.

Supplementary file1 (DOCX 15 kb)

## Data Availability

The dataset used and analyzed during the current study is available from the corresponding author on reasonable request.
