# Supplementary material for: Dyspnea and dystussia in Parkinson’s disease: patient-reported prevalence and determinants
Source: J Neurol. 2025 Mar 22;272(4):283. doi: 10.1007/s00415-025-13008-0 (PMC11929639; doi:10.1007/s00415-025-13008-0)
Supplement: Supplementary file 1 — Supplementary file1 (DOCX 15 kb) [file 415_2025_13008_MOESM1_ESM.docx]

**Supplementary data**

**S1: PCA analysis**

Correlation matrix of respiratory dysfunction

| **Correlation Matrix^a,b^** | | | | | | | |
| --- | --- | --- | --- | --- | --- | --- | --- |
|  | | Probl ademhaling | Ademtekort | Benauwd gevoel | Vaak keel schrapen | Vaak hoesten | Problemen ophoesten |
| Correlation | Probl ademhaling | 1,000 | ,735 | ,556 | ,204 | ,246 | ,240 |
|  | Ademtekort | ,735 | 1,000 | ,498 | ,157 | ,227 | ,152 |
|  | Benauwd gevoel | ,556 | ,498 | 1,000 | ,208 | ,186 | ,228 |
|  | Vaak keel schrapen | ,204 | ,157 | ,208 | 1,000 | ,479 | ,432 |
|  | Vaak hoesten | ,246 | ,227 | ,186 | ,479 | 1,000 | ,443 |
|  | Problemen ophoesten | ,240 | ,152 | ,228 | ,432 | ,443 | 1,000 |
| a. Imputation Number = 10 | | | | | | | |
| b. Determinant = ,157 | | | | | | | |

This table shows the correlation matrix between the six respiratory symptoms.

| **KMO and Bartlett's Test^a^** | | |
| --- | --- | --- |
| Kaiser-Meyer-Olkin Measure of Sampling Adequacy. | | ,719 |
| Bartlett's Test of Sphericity | Approx. Chi-Square | 1734,009 |
|  | df | 15 |
|  | Sig. | ,000 |
| a. Imputation Number = 10 | | |

| **Total Variance Explained^a^** | | | | | | | |
| --- | --- | --- | --- | --- | --- | --- | --- |
| Component | Initial Eigenvalues | | | Extraction Sums of Squared Loadings | | | Rotation Sums of Squared Loadings^b^ |
|  | Total | % of Variance | Cumulative % | Total | % of Variance | Cumulative % | Total |
| 1 | 2,685 | 44,754 | 44,754 | 2,685 | 44,754 | 44,754 | 2,373 |
| 2 | 1,422 | 23,695 | 68,449 | 1,422 | 23,695 | 68,449 | 2,104 |
| 3 | ,603 | 10,044 | 78,492 |  |  |  |  |
| 4 | ,557 | 9,277 | 87,769 |  |  |  |  |
| 5 | ,478 | 7,968 | 95,737 |  |  |  |  |
| 6 | ,256 | 4,263 | 100,000 |  |  |  |  |
| Extraction Method: Principal Component Analysis. | | | | | | | |
| a. Imputation Number = 10 | | | | | | | |
| b. When components are correlated, sums of squared loadings cannot be added to obtain a total variance. | | | | | | | |
